# Cultural Considerations: Pharmacological and Nonpharmacological Means for Improving Blood Pressure Control among Hispanic Patients

**DOI:** 10.1155/2012/831016

**Published:** 2011-10-20

**Authors:** Neela K. Patel, Robert C. Wood, David V. Espino

**Affiliations:** ^1^Division of Geriatrics, Department of Family and Community Medicine, University of Texas Health Science Center San Antonio, San Antonio, TX 78229, USA; ^2^Department of Family and Community Medicine, University of Texas Health Science Center San Antonio, San Antonio, TX 78229, USA

## Abstract

Cardiovascular disease is a leading cause of morbidity and mortality in the United States, and its prevention and treatment remain a priority for the medical community. Ethnic variations account for some differences in the prevalence of hypertension and blood pressure (BP) control rates among Hispanics, indicating the need for culturally appropriate management models. Aggressive treatment strategies are key to achieving optimal BP control in high-risk Hispanic patients. Hypertension in this ethnic group continues to be a major health concern. Of note, when provided access to comprehensive care, Hispanics demonstrate similar response rates to treatment as the majority of non-Hispanic whites. This highlights the importance of effective, culturally responsive hypertension management among high-risk Hispanic patients for achieving observable, positive health outcomes.

## 1. Introduction

In the United States, cardiovascular disease is a leading cause of morbidity and mortality; therefore, prevention and treatment remain a priority for the medical community [1]. Though each ethnic group and segments of the populations are affected by cardiovascular disease, it has been observed that socioeconomic and racial factors are strongly correlated with health. Hypertension awareness often accounts for minor differences in blood pressure (BP) control between Hispanics and non-Hispanics, resulting in the need for culturally appropriate education and management models [2].

## 2. Acculturation, Language, and Hypertension

In the United States, researchers note that factors such as modernization, education, and structural assimilation were correlated with favorable BP profiles [3]. The prevalence of hypertension among Hispanic Americans appears to increase with the process of acculturation and is inversely correlated with socioeconomic status [4]. Indeed, acculturation and language proficiency in this ethnic group can be directly correlated with the incidence of diabetes and associated morbidities, which have implications for cardiovascular health [5]. 

For example, among Mexican Americans, acculturation and age are strong predictors of hypertension as opposed to economic status [6]. Based on these findings, Mexican American women who are English proficient and had healthcare coverage were more likely to be screened for heart disease [7]. This assertion is confirmed by Sundquist and Winkleby [8], who found that risk of cardiovascular disease is highest for US-born Spanish-speaking individuals, intermediate for US-born English-speaking individuals, and lowest for individuals of Mexican origin. In spite of increased awareness on the part of both patients and their primary care providers, Spanish-speaking patients continue to have higher rates of hypertension, LDL cholesterol, and fasting blood glucose, when compared to acculturated, or English-speaking Hispanics [9]. US-born Mexican Americans are more likely to obtain BP screenings than their Mexican counterparts, highlighting the need for clinicians to carefully note a patients' country of origin and other sociodemographic risk factors, such as lower levels of education [10].

## 3. Hispanic Identity

Hispanic and Latino individuals are the largest minority in the US population at over 15 percent. This ethnic group exhibits a great degree of genetic, physiologic, and socioeconomic variability. 

Due to the complexity of this group, intracultural variability for the treatment of hypertension in Hispanics deems greater appreciation. Findings suggest that within this “single” ethnic group, there are differences in disease prevalence and complications and in access to health care. For example, Getaneh et al. [11] report that Dominican Americans have a higher incidence of diabetes than Mexican Americans. 

Lin et al. [12] reported that the incidence of hypertension was 66 percent among Puerto Rican men and 73 percent among Dominican men, compared with 69 percent for non-Hispanic white men. Also, Hispanic Americans of Caribbean descent have a hypertension profile similar to that of African Americans [13].

## 4. Prevention and Patient Awareness

Awareness is a key in combating hypertension, which is often described as the “silent disease.” It has been found that Mexican American hypertensive subjects had significantly poorer BP control than non-Hispanic white hypertensive subjects. This trend is often attributed to low hypertension awareness among Hispanics when compared with their counterparts in other ethnic groups [13]. 

The most effective way to ensure good health is to emphasize a healthy, preventative lifestyle. Prevention behaviors can be taught in childhood and actively practiced through adolescence and into young adulthood. Increasingly, adolescents are at risk for developing insulin resistance and worsened cardiovascular disease risk factors [14]. 

Such risk factors set the stage for obesity, which is placing young people at risk for diabetes and hypertension [14]. Hispanics have been shown to have a 21 percent greater prevalence of obesity compared with non-Hispanic whites [15]. This trend among adolescents suggests prevention and health literacy beginning should be emphasized at a very early age by way of school-based and community-based prevention strategies. Such strategies can directly impact health outcomes and academic performance [16]. Because diabetes tends to be diagnosed in Mexican Americans at younger ages, it is important to screen this population at earlier timepoints (i.e., adolescence) for hypertension. In addition, African Americans and Mexican Americans have the lowest rates of BP control though under treatment [17]. Specifically, it has been reported that among patients with hypertension, medication use was lower in Hispanics (45%) than in non-Hispanic whites (54%) [18]. These high rates indicated a need for educational campaigns to raise awareness.

## 5. Incidence of Hypertension in Hispanics

Although historically, Mexican Americans' prevalence for heart disease and hypertension has been lower than among their counterparts in the general population, there has been an increase since 1993 making it the leading cause of death among Mexican Americans [10, 19]. This increasing incidence is partly indicative of a failure to prevent cardiovascular disease through management and education [20].

Although BP control and cholesterol levels have improved for adults with cardiovascular disease and diabetes, in general, differences in disease control with respect to ethnicity and age have not decreased, with hypertension control remaining higher among non-Hispanic whites [21]. This demonstrates that, with respect to demographic trends, the overall incidence of hypertension among Mexican Americans continues to be a matter of concern for public health, with severely adverse outcomes seen in 6 percent of the Mexican American older population [22].

## 6. Medications and Treatment

The findings from ALLHAT (antihypertensive lipid lowering treatment to prevent heart attack trial) demonstrate that Hispanic participants had equivalent or superior BP control compared with non-Hispanics in a clinical trial setting where patients with hypertension possessed equal access to medical care. In this trial, medication was also provided at no cost. Compared with non-Hispanic whites, Hispanic whites had a 20 percent increased likelihood of achieving BP control than non-Hispanic whites, after adjusting for multiple factors that predict BP control [23].

Hispanic Americans have higher rates of cardiac morbidity. As a result, researchers have suggested that angiotensin-converting enzyme inhibitors (ACEIn) and angiotensin II receptor blockers (ARBs) may be particularly useful in the Hispanic population owing to the ability of RAAS inhibitors to protect against end-organ damage caused by hypertension [24]. 

The effectiveness of proper medication regimens should not be underestimated among Hispanic patients. It has been noted that the use of antihypertensive regimens resulted in superior BP control and fewer cardiovascular events in Hispanic women compared with non-Hispanic white women [25]. Furthermore, it has been found that adherence to treatment was better among blacks (83.7%) and Hispanics (83%) than among whites (78.4%) [26]. 

Several large trials continue to explore this important line of inquiry. The ongoing telmisartan alone and in combination with ramipril global endpoint trial (ONTARGET) [27] study suggests that high-risk patients benefit from combination therapy. In the INVEST (international verapamil-trandolapril study) trial, no significant differences were noted between verapamil and atenolol-based strategies for the primary outcome of death and nonfatal myocardial infarction or stroke by ethnicity [28]. The INVEST researchers discovered that Hispanics, in particular, had a lower risk of adverse cardiovascular outcomes than non-Hispanics. A third study, the VALUE (valsartan long-term use evaluation) trial, found no differences by ethnicity between valsartan- and amlodipine-based therapies for the composite cardiac outcome [29]. Moreover, the INCLUSIVE study demonstrated that combination therapy led to substantial decreases in systolic BP among Hispanic patients [30].

The efficacy of the ACE and ARB combinations has been demonstrated in many studies. ACE and ARB agents each have their own pharmacologically relevant profiles, including different efficacy profiles for BP control [31]. There may be unwanted side effects, such as angioedema, which may be associated with some ARBs [32].

Some studies have suggested that primary care providers should aim for more aggressive treatment of their hypertensive patients, regardless of ethnicity [26]. Compliance with JNC prescribing guidelines and more aggressive increase of medication dosages when indicated can improve rates of hypertension control and reduce the racial differences in hypertension outcomes [26]. Overall, the control rates of hypertension among Mexican Americans have increased significantly over a period of 5 years and have even been described as “encouraging” [19].

## 7. Comorbidities and Quality of Life in Hispanics with Hypertension

Comorbidity rates and mortality related to hypertension may be higher among individuals in the Hispanic community. For elderly patients, hypertension and its associated comorbidities present special challenges. It has been found that there is a direct correlation between age, the severity of the hypertension, and cognitive decline among Mexican Americans aged 65 years or older [33]. Lower extremity functional limitation (LEFL) is one of the comorbidities associated with suboptimal prescribing in hypertension [34]. In addition, it has also been noted that psychological effects caused by hypertension and the need for daily management can lead to a decreased quality of life [35]. 

## 8. Choosing the Right Treatments for Hypertension and Comorbidity Management

Targeted efforts to increase the use of ACE inhibitors or ARBs have been shown to improve quality of care for high-risk patients, regardless of ethnicity.

Several practical clinical management strategies have been found effective in improving clinical outcomes. For example, because nonadherence was found to be correlated with having BP checked in an emergency room, changing the locus of care for hypertension from emergency rooms to primary care may improve outcomes [36]. Another strategy, the dietary approaches to stop hypertension (DASH) combination diet, may be an effective strategy for preventing and treating hypertension, particularly in high-risk populations [37].

## 9. Special Considerations for Hispanic Women

It has been demonstrated that Hispanic women exhibit less favorable risk profiles for hypertension than non-Hispanic women [38]. Among younger, perimenopausal women, African American and Hispanic women exhibited the lowest rates of control for hypertensive risk factors [39], and hypertension control rates continue to be subobtimal for Hispanic women aged 60 years or older [40]. However, Hispanic women continue to have better treatment and control rates for hypertension than Hispanic men (Table 1).

## 10. Promotoras and Community Health Workers

The Hispanic community has a tradition of relying upon community health workers, also known as promotoras, to help improve the health of individuals in the community. For example, The Salud para su Corazón-HRSA initiative was successful in the development of infrastructure directed at supported promotoras de salud workforce in the US-Mexico border region [41]. Reductions in salt and cholesterol intake were shown to improve through promotora intervention [41]. A diabetes self-management education program can be effectively implemented in community settings for glycemic control [42]. In looking at community-based prevention, intervention was found to significantly increase the number of women meeting national recommendations for fruit and vegetable consumption [43].

Community-health workers also play a vital role in addressing the needs of rural patients. Optimization of healthcare delivery is needed for Hispanics who live in rural areas and who tend to be at higher risk for hypertension and less responsive to primary and specialty care compared with their urban counterparts [44]. The delivery of rural healthcare to patients with hypertension requires new strategies such as programs targeting therapeutic inertia, home-based monitoring of BP, and internet-based communication programs [45]. 

## 11. Innovative Strategies for Treatment and Prevention

Continuing care and lifestyle modification are practical, effective means of controlling hypertension among Hispanics. It has been shown that effective, culturally appropriate interventions result in increased adherence to lifestyle and diet modification [46].

One approach that has been suggested is a community-academic partnership, which enabled the successful recruitment, intervention, and assessment of Hispanics at risk for diabetes [47]. While this approach was adopted with the goal of reducing the risk of diabetes, its methods can perhaps be applied to patients with hypertension to achieve BP control. Another suggested intervention, the use of senior centers as an intervention point for offering behavioral counseling and implementing lifestyle changes, has resulted in reduced systolic BP and increased adherence [48]. Church-based interventions may be a way to reduce stroke [49]. Culturally appropriate community-based interventions, such as those designed to engage individuals in physical activity while also increasing their awareness of risk factors, can impact clinical outcomes [50]. Cultural adaptation strategies, such as Spanish-language intervention models leading to lifestyle-based management programs, may also be effective [51]. 

Family-based interventions to raise awareness about hypertension can be effective, especially if they are administered through school-based programs that can educate both parents and children. In fact, it has been demonstrated that school-based programs are effective and correlated with reductions in BP among adult family members [52].

Nursing care management programs can also be clinically effective as well as cost effective in a medically indigent, culturally diverse population and provide an immediate benefit to a high-risk population [53]. Pharmacists play an important role in community-based screenings and in identifying high-risk individuals for cardiac disease [54]. To combat the increasing risk of hypertension and diabetes in increasingly younger groups, school-based interventions have been developed and implemented in Southwestern states [55]. 

## 12. Conclusions

Innovative, aggressive, timely treatment, with support in a community-based context, is needed. Interventions to reduce disparities in cardiovascular outcomes should consider the need to intensify drug therapy among all high-risk populations, as the aggressiveness of treatment strategies is key to achieving optimal BP control in high-risk patients. Although hypertension continues to be a major health concern for Hispanics, when these patients are given access to effective care, they demonstrate the same responsiveness to treatments as the majority of non-Hispanic white Americans.

These findings highlight the importance of effective, culturally responsive hypertension management in high-risk Hispanic patients for achieving observable, positive health outcomes.

## Figures and Tables

**Table 1:** Percentage of noninstitutionalized US adults with hypertension* and, among those with hypertension, estimated percentage of persons who are aware of^‡^, treated for^§^, and in control of their condition^¶^, by sex, race/ethnicity, and age group—United States, 1999–2002.

Characteristic**	Hypertension prevalence % (95% CI^‡‡^)	Awareness of condition % (95% CI)	Under current treatment % (95% CI)	Condition controlled % (95% CI)
Sex				
Men	27.8 (24.9–29.7)	59.4 (55.8–63.1)	45.2 (40.9–49.6)	27.5 (23.7–31.3)
Women	29.0 (27.3–30.8)	69.3 (61.7–77.0)	56.1 (29.2–63.1)	35.5 (28.4–42.7)
Race/Ethnicity				
White, non-Hispanic	27.4 (25.3–29.5)	62.9 (57.3–68.5)	48.6 (44.1–53.1)	29.8 (25.7–34.0)
Black, non-Hispanic	40.5 (38.2–42.9)	70.3 (64.9–75.9)	55.4 (51.2–59.6)	29.8 (25.2–34.5)
Mexican American	25.1 (23.1–27.1)	49.8 (40.4–59.2)	34.9 (27.5–42.3)	17.3 (10.7–23.8)
Age group (yrs)				
20–39	6.7 (5.3–8.2)	48.7 (38.8–58.7)	28.1 (20.1–36.1)	17.6 (11.6–23.7)
40–59	29.1 (25.9–32.4)	73.5 (69.1–77.90	61.2 (57.1–65.2)	40.5 (36.4–44.5)
≥60	65.2 (62.4–68.0)	72.4 (70.0–74.7)	65.6 (61.9–69.3)	31.4 (28.7–34.2)

Total^¶¶^	**28.6 (26.8**–**30.4)**	**63.4 (59.4**–**67.4)**	**45.3 (45.3**–**52.8)**	**29.3 (26.0**–**32.7)**

*Had a blood pressure measurement ≥140 mm Hg systolic or ≥90 mm Hg diastolic or took antihypertensive medication.

^‡^Told by a health-care professional that blood pressure was high.

^§^Took antihypertensive medication.

^¶^Hypertension levels <140 mm Hg systolic and <90 mm Hg diastolic.

**All characteristic estimates (excluding age group) are age adjusted [15].
